# *Tbx6* controls left-right asymmetry through regulation of *Gdf1*

**DOI:** 10.1242/bio.032565

**Published:** 2018-04-12

**Authors:** Daniel Concepcion, Hiroshi Hamada, Virginia E. Papaioannou

**Affiliations:** 1 Department of Genetics and Development, Columbia University Medical Center, New York, NY 10032, USA; 2 RIKEN Center for Biosystems Dynamics Research, Kobe, 650-0047, Japan

**Keywords:** *Tbx6*, *Gdf1*, Left-right asymmetry, Mouse development, Axis determination

## Abstract

The Tbx6 transcription factor plays multiple roles during gastrulation, somite formation and body axis determination. One of the notable features of the *Tbx6* homozygous mutant phenotype is randomization of left/right axis determination. Cilia of the node are morphologically abnormal, leading to the hypothesis that disrupted nodal flow is the cause of the laterality defect. However, Tbx6 is expressed around but not in the node, leading to uncertainty as to the mechanism of this effect. In this study, we have examined the molecular characteristics of the node and the genetic cascade determining left/right axis determination. We found evidence that a leftward nodal flow is generated in *Tbx6* homozygous mutants despite the cilia defect, establishing the initial asymmetric gene expression in *Dand5* around the node, but that the transduction of the signal from the node to the left lateral plate mesoderm is disrupted due to lack of expression of the Nodal coligand Gdf1 around the node. *Gdf1* was shown to be a downstream target of Tbx6 and a *Gdf1* transgene partially rescues the laterality defect.

## Abstract (precis)

**Summary:** Tbx6 affects morphology of the cilia of the node, but a leftward nodal flow is still generated. Downstream of nodal flow, Tbx6 regulates the Nodal coligand Gdf1 leading to disruption of left/right axis determination.

## INTRODUCTION

The bilateral symmetry of the early mouse embryo is broken during early gastrulation when genes are asymmetrically expressed around the embryonic node and in the lateral plate mesoderm (LPM). The earliest known morphological left-right asymmetry in the mouse is the leftward displacement of the future atrioventricular canal, followed by the dextral looping of the linear heart tube. As development proceeds, the embryo undergoes a process of axial rotation towards its right side and additional morphological asymmetries arise in virtually all internal organ systems (Beddington and Robertson, 1999; Brown and Anderson, 1999; Levin et al., 1995; Miller and White, 1998). The initial asymmetric gene expression around the node is driven by the motility of the monocilia on cells of the node, which creates a leftward flow of extracellular fluid. This flow is sensed by the cilia of the crown cells on the left side of the node through a mechanism involving the ion channel gene *Pkd2*, initiating a cascade of asymmetric gene expression (Babu and Roy, 2013; Delling et al., 2016; Norris, 2012). *Dand5* (also known as *Cerl-2*), a Nodal antagonist, is more highly expressed on the right side, while *Nodal*, which is initially expressed symmetrically around the node, becomes more highly expressed on the left (Hamada et al., 2002; Krebs et al., 2003). Nodal signal then spreads to the left LPM where it induces its own expression and that of other asymmetric genes such as *Pitx2* (Norris, 2012). Perinodal expression of the Nodal co-ligand, *Gdf1* is essential for the long-range transfer of the Nodal signal from the node to the left LPM (Tanaka et al., 2007). Homozygous null mutants for either *Nodal* or *Gdf1* show a loss of expression of left-specific genes in the LPM (Rankin et al., 2000; Saijoh et al., 2003).

This study focuses on the T-box gene *Tbx6* that is expressed between embryonic day (E) 6.5 and E13.5 in the primitive streak, the presomitic mesoderm and the tail bud, but not in the node, although node precursors do express the gene (Concepcion et al., 2017). *Tbx6* null mutants die around E9.5 with defects in anterior somite patterning, ectopic neural tubes and an enlarged tail bud (Chapman et al., 1996; Chapman and Papaioannou, 1998; Takemoto et al., 2011). They display heterotaxia with randomized direction of embryo turning and heart looping and have defects in node and cilia morphology. *Dll1* and *Nodal* expression is reduced in the perinodal region and most mutant embryos show an absence of expression of *Nodal*, *Pitx2* and *Lefty2* in the LPM as well as a lack of Ca^+2^ signaling at the node (Hadjantonakis et al., 2008). The mechanism by which *Tbx6* affects the development of the node and node cilia and how this affects the expression of left-right specific genes in the LPM is not fully understood. In this study we found that despite the severe defect in nodal cilia, there appears to be a functional leftward nodal flow and the main effect of *Tbx6* on left-right axis determination is downstream of nodal flow and the detection of the flow by perinodal crown cells. Furthermore, we identified *Gdf1* as a downstream target of Tbx6 in the transduction of the perinodal Nodal signal to the LPM.

## RESULTS

### Node development in *Tbx6* homozygous mutants

**Fig. 1.**
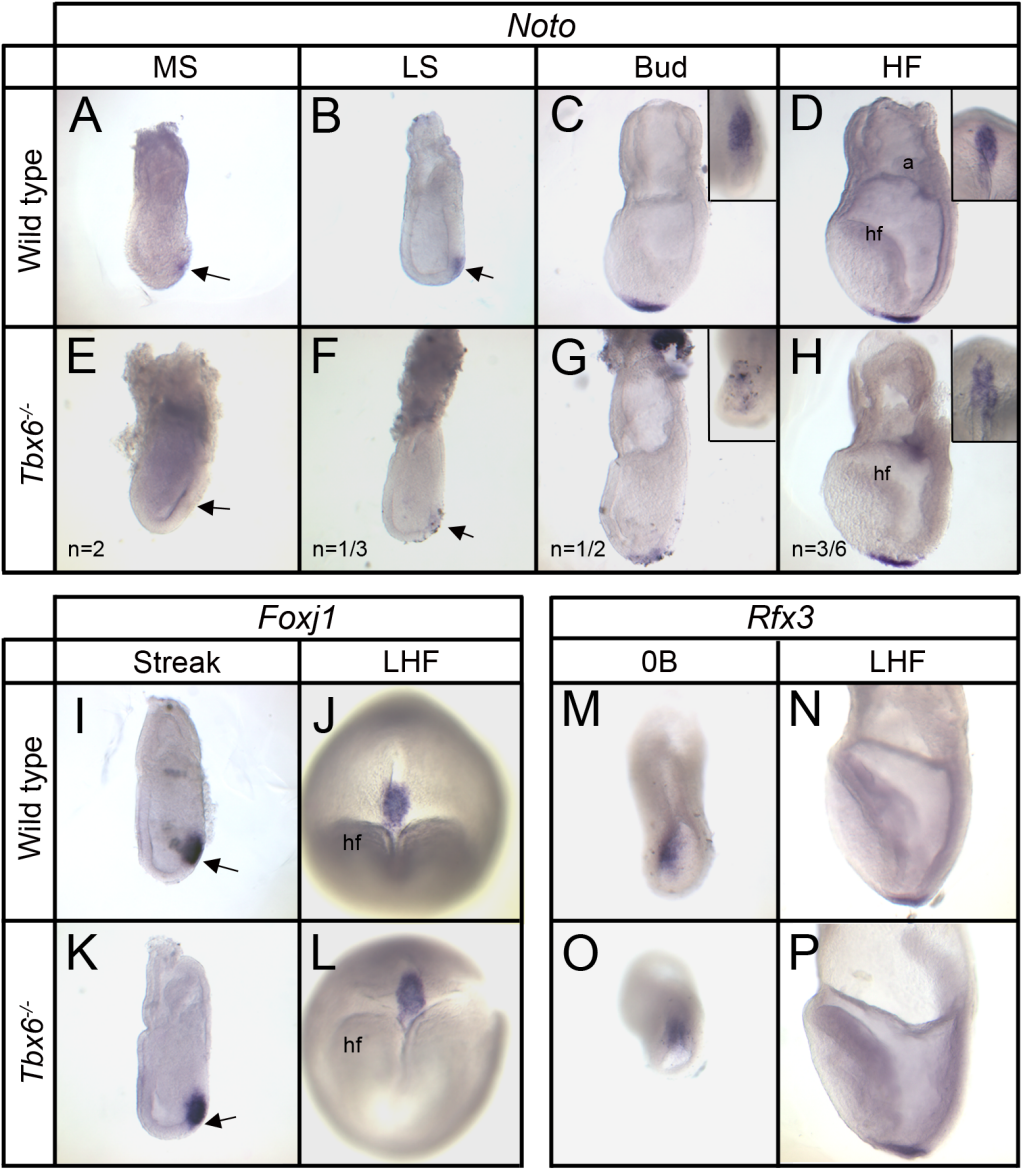
**Expression of transcription factors involved in node and cilia formation in wild-type and *Tbx6* homozygous mutant embryos.** (A-H) Expression of *Noto* in wild-type (A-D) and *Tbx6* mutant (E-H) embryos from midstreak to head-fold stages. A-H are left lateral views and the insets in C,D,G,H are ventral views of the distal tip of the corresponding embryo with anterior at the bottom. Normal early expression is in the anterior primitive streak and later in the node and nascent notochord whereas expression in *Tbx6* mutants is delayed and less uniform. ‘*n*=’ indicates the proportion of mutant embryos with the illustrated expression pattern, while the remainder was similar to wild type. (I-L) Expression of *Foxj1* in wild-type (I,J) and mutant (K,L) embryos from early streak stage (lateral views) and late head-fold stage (ventral view with anterior at bottom) showing similar expression patterns. (M-P) Expression of *Rfx3* in wild-type (M,N) and *Tbx6* mutants (O,P) at 0 bud and late head-fold stages showing similar expression patterns. M,N,P are left lateral views; O is a ventral view with anterior at the bottom. Arrows indicate anterior end of the primitive streak. a, allantois; Bud, allantoic bud stage; HF, head-fold stage; hf, head fold; LHF, late head-fold stage; LS, late streak stage; MS, midstreak stage; 0B, zero bud stage.

In trying to understand how *Tbx6* affects the development of the nodal cilia, we examined the expression of several transcription factor genes known to affect node or cilia development and left-right axis determination. *Noto* is expressed at the anterior end of the primitive streak in precursors of the embryonic node and later in the node and developing notochord (Fig. 1A-D) (Beckers et al., 2007). *Tbx6* homozygotes have no detectable *Noto* expression at the midstreak stage (Fig. 1E); at late streak, allantoic bud and head-fold stages, half of the mutants had weak, punctate *Noto* expression (Fig. 1F-H), while the others showed normal expression. Thus, in stage-matched embryos, more than half of the mutants (7/13) have delayed or irregular *Noto* expression in the developing node compared with wild-type embryos.

In contrast, mutant (*n*=16) and wild-type embryos showed similar *Foxj1* expression in precursors of the node at the streak stage and in the node through early somite stages (Fig. 1I-L and not shown) (Brody et al., 2000), with the exception of a single mutant with an irregular pattern at the early bud stage. *Rfx3* expression in the embryonic node between 0 bud and head-fold stages was indistinguishable between wild type and mutants (*n*=15) (Fig. 1M-P) (Bonnafe et al., 2004).

Several lines of evidence implicate non-canonical Wnt signaling in the determination of left-right asymmetry and node development in vertebrates (Kitajima et al., 2013; Minegishi et al., 2017; Oishi et al., 2006; Park et al., 2006; Zhang and Levin, 2009). We investigated the expression of the non-canonical Wnt signaling ligands *Wnt5a* and *Wnt5b*, and the receptors *Fzd2*, *Fzd3* and *Fzd10*, all of which overlap to some extent with *Tbx6* expression from primitive streak to early somite stages. *Tbx6* homozygous mutant embryos showed normal expression of all of these components of the non-canonical Wnt signaling pathway (Fig. S1).

### Is there functional nodal flow in *Tbx6* mutant embryos?

**Fig. 2.**
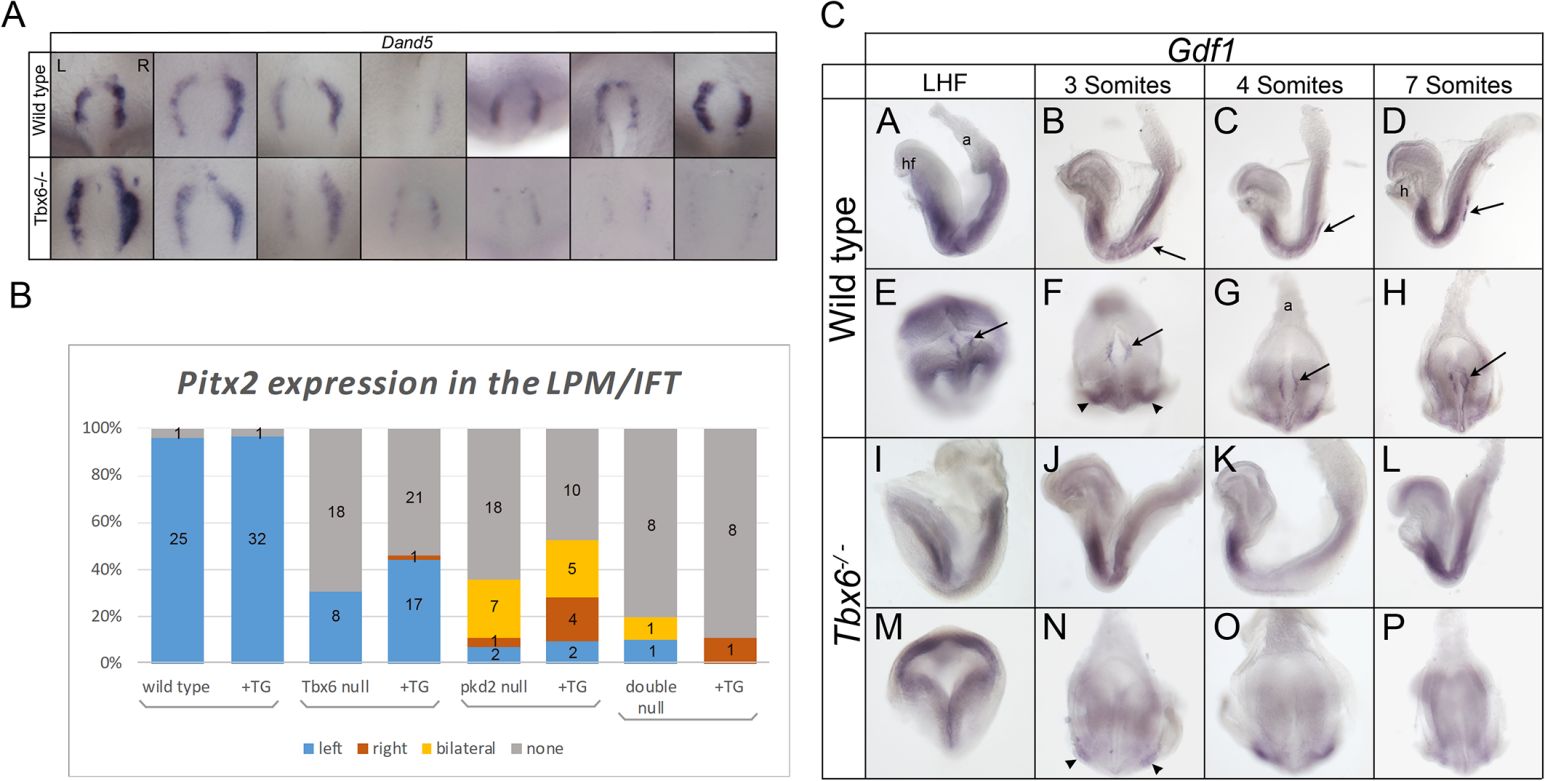
**Asymmetric gene expression in *Tbx6* mutant embryos, lack of perinodal *Gdf1* expression and rescue by *Gdf1* transgene.** (A) Perinodal expression of *Dand5* in wild-type and *Tbx6* homozygous mutant embryos. Expression is more intense on the right side of the node in at least four out of seven wild-type embryos and in all but one of the mutant embryos (far right) (*P*>0.05; Fisher’s exact test). An additional mutant embryo had no visible expression and is not shown. All panels are ventral views with anterior to the bottom of the panel. L, left side; R, right side. (B) Graphical representation of the proportion of embryos expressing *Pitx2* in the LPM/IFT of embryos of the indicated genotypes, with (+TG) or without the *Gdf1 node-Tg*. Numbers in the bars are the *n* values for each category. (C) Expression of *Gdf1* in wild-type (A-H) and *Tbx6* homozygous mutant embryos (I-P) between late head-fold (LHF) and seven somite stages. Left lateral (A-D,I-L) and ventral (E-H,M-P; anterior at the bottom) views showing *Gdf1* expression in the perinodal region of wild-type embryos at LHF stage and bilaterally in the lateral plate mesoderm and perinodal region at somite stages, whereas mutant embryos lack perinodal expression at all stages (*n*=12). Arrows point to expression in the perinodal region; arrowheads point to expression in the LPM. a, allantois; h, heart; hf, head folds.

In our previous study, we observed reduced *Nodal* expression around the node but did not draw conclusions about asymmetry of expression due to the low level of expression observed (Hadjantonakis et al., 2008). Thus it remained an open question whether an asymmetric signal was present at the node. As an alternative readout of nodal flow, we examined the perinodal expression of *Dand5* and found that expression appeared to be greater on the right side of the node in 7/8 mutants, with undetectable expression in the eighth embryo (Fig. 2A). Although the number of embryos is small, this was the first indication that a directional nodal flow is detected by the crown cells in spite of abnormal cilia and the apparent absence of perinodal intracellular Ca^2+^ signaling in *Tbx6* mutant embryos (Hadjantonakis et al., 2008).

To investigate this phenomenon further and to ascertain whether the asymmetric perinodal signal was mediated through the crown cell cilia in the established pathway, we made use of a null mutation in *Pkd2*, a calcium permeable ion channel gene shown to be necessary for the perinodal cilia to sense the nodal flow and initiate asymmetric gene expression (Pennekamp et al., 2002; Yoshiba et al., 2012). We produced compound *Tbx6*; *Pkd2* mutants and assessed embryos for expression of *Pitx2* in the LPM of 2-7 somite-stage embryos or inflow tract (IFT) of the heart of more advanced embryos. The majority of *Tbx6* mutants (18/26) had no *Pitx2* expression in the LPM/IFT, whereas the remainder (8/26) had left-sided expression (Fig. 2B; Table S1). Most *Pkd2* mutants had no or bilateral expression (18/28 and 7/28, respectively), while two had left-sided and one had right-sided expression (see Fig. S2). The majority of double-homozygous *Tbx6; Pkd2* mutants lacked *Pitx2* expression (8/10) with one embryo showing left-sided and one showing bilateral expression.

Additional *Tbx6*; *Pkd2* double homozygotes from this same breeding cohort that also carried a transgene for *Gdf1* (+TG in Fig. 2B; see later section; Table S1) had no (8/9) or right-sided (1/9) expression. Taken together, the double homozygotes do not show a bias toward left-sided expression (1/19), as do the Tbx6 homozygous mutants that show sporadic *Pitx2* expression (8/8), leading to the conclusion that the effect of *Tbx6* on left-biased expression of *Pitx2* is hypostatic to the effect of *Pkd2*, as would be expected on the basis of the generally accepted pathway for detection of the nodal flow. The fact that sporadic expression of *Pitx2* in *Tbx6* mutants is left-sided rather than random further supports the conclusion from *Dand5* expression that there is a functional leftward nodal flow.

### Tbx6 is upstream of *Gdf1*

With evidence for an asymmetric signal at the node of Tbx6 mutant embryos mediated through detection of a nodal flow by perinodal cilia, we investigated the next step in the cascade by examining expression of *Gdf1*, which is essential for transfer of the perinodal Nodal signal to the left LPM. At the LHF stage, wild-type embryos express *Gdf1* throughout the embryo with discrete bilateral expression in the perinodal region. Between zero and seven somite stages, *Gdf1* is expressed bilaterally in both the perinodal region and LPM (Fig. 2C: B-D,F-H) (Rankin et al., 2000). In *Tbx6* mutant embryos, *Gdf1* is expressed uniformly throughout the embryo at the LHF stage (*n*=2) and bilaterally in the LPM at later stages (*n*=10); however, no signal is observed in the perinodal region at any stage (Fig. 2C: I-P), leading to the hypothesis that *Tbx6* regulates *Gdf1* in the perinodal region.

**Fig. 3.**
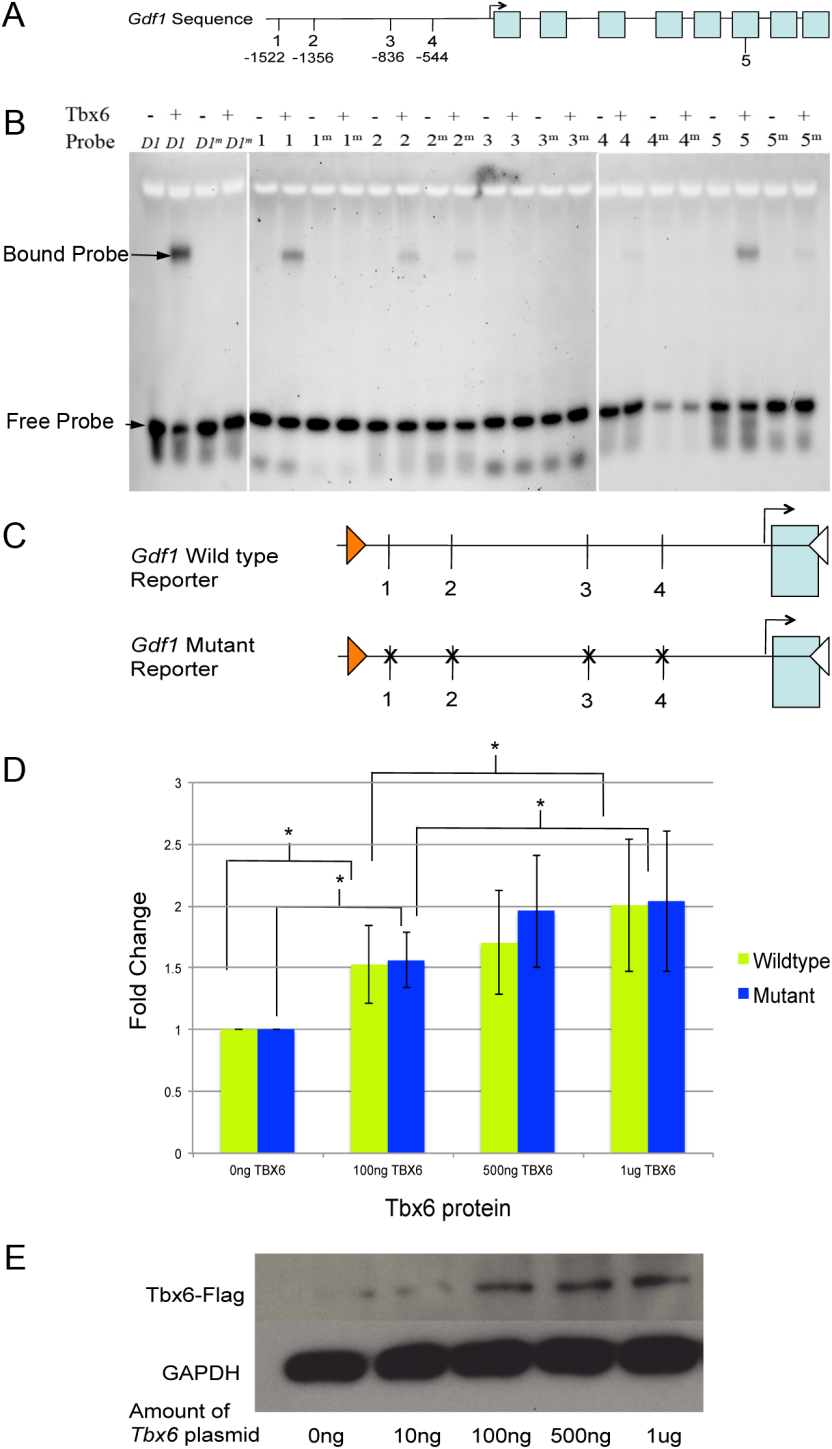
**Characterization of *Tbx6* putative binding sites in the *Gdf1* promoter region by EMSA and luciferase assays.** (A) Schematic of *Gdf1* genomic region and locations of putative *Tbx6* binding sites relative to the transcriptional start site of *Gdf1*. Blue boxes represent exons. (B) EMSA with wild-type and mutant (m) DNA probes for each of five *Tbx6* putative binding sites run with or without (+ or −) Tbx6 protein. A known *Tbx6* binding site of *Dll1* (*D1*) was used as a positive binding control. (C) Diagram of wild-type and mutant *Gdf1* luciferase reporters with relative locations of putative *Tbx6* binding sites. ‘X’s represents a mutated site. (D) The bar graph represents the relative fold change of luciferase reporter activity with increasing amount of Tbx6 protein for the wild-type and mutant reporter. Asterisks indicates a significant difference between groups indicated (ANOVA). (E) Western blot for FLAG-tagged Tbx6 with an anti-FLAG antibody.

Using Conservation-Aided Transcription-Factor-Binding Site Finder (COTRASIF; http://biomed.org.ua/COTRASIF/) with a position weight matrix for Tbx6 to scan 2 kb upstream and downstream of the transcriptional start site of *Gdf1*, we found five putative *Tbx6* binding sites (Fig. 3A; Table S2). Electrophoretic mobility shift assays (EMSA) were used to test whether Tbx6 protein binds to fluorescently labeled DNA probes for each of the five sites *in vitro*. Probes with mutated sites (Table S2) were used to test binding specificity. Results show that Tbx6 protein can bind specifically to sites #1, 4, and 5. Site #3 showed no binding and site #2 showed non-specific binding in which both the wild-type and mutated probe bound to Tbx6 protein (Fig. 3B).

Luciferase assays were used to test whether *Tbx6* can regulate gene expression and whether it does so through the putative binding sites in the *Gdf1* promoter. NIH 3T3 cells were transfected with a *Tbx6* expression construct and a luciferase reporter containing 2.3 kb of the *Gdf1* promoter region or the *Gdf1* promoter region with mutated putative Tbx6 binding sites (excluding site #5) (Fig. 3C). Transfection of increasing amount of Tbx6 expression construct leads to a twofold increase in luciferase reporter activity. However, no difference in activity between wild-type and mutant luciferase reporters was observed (Fig. 3D). Western blot demonstrates that transfected cells express Tbx6 protein (Fig. 3E). Thus while Tbx6 regulates luciferase activity from the *Gdf1* promoter, it does not do so through these putative binding sites.

Embryos homozygous mutant for either *Tbx6* or *Gdf1* have randomization of heart looping and embryo turning, while heterozygous embryos have normal situs (Hadjantonakis et al., 2008; Rankin et al., 2000). To determine the extent of interaction between *Tbx6* and *Gdf1* we crossed *Tbx6* and *Gdf1* heterozygous mice to produce double heterozygotes. Normal direction of heart looping and embryo turning was observed in all *Tbx6^+/−^;Gdf1^+/−^* double heterozygous embryos (*n*=21). For a more sensitized screen, the dose of *Tbx6* was further reduced using a hypomorphic allele, *Tbx6^rv^*, (Watabe-Rudolph et al., 2002) in combination with the null allele. *Tbx6^rv/−^;Gdf1^+/−^* mutant embryos also had normal situs at E9.5 (*n*=11).

### *Gdf1* perinodal expression in *Tbx6^−/−^* mutants rescues asymmetric expression of Pitx2

**Fig. 4.**
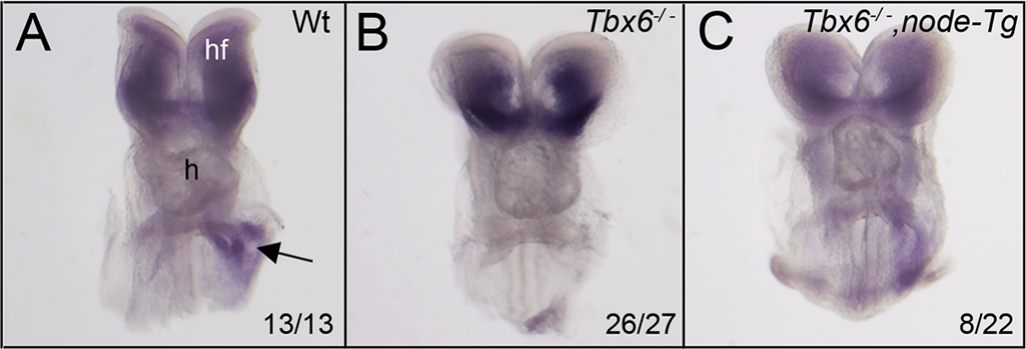
**Expression of *Pitx2* in *Tbx6^−/−^*, *node-Tg* mutant embryos at E8.5.** Frontal views show *Pitx2* expression in the head folds of all embryos (A-C) and in the left inflow tract of wild-type (A) and some *Tbx6^−/−^; node-Tg* embryos (C), but not in most *Tbx6^−/−^* embryos (B). Numbers indicate the proportion of embryos with the expression pattern shown. h, heart; hf, head folds.

We made use of a transgene, *node-Tg*, that expresses the cDNA of *Gdf1* bilaterally in the perinodal region to restore *Gdf1* expression in *Tbx6* homozygous mutants. In the first series of experiments crossing the Tbx6 mutant stock with mice carrying the Gdf1 transgene, all wild-type embryos at E8.5 showed expression of *Pitx2* in the left IFT of the heart (*n*=13), whereas the majority of *Tbx6* homozygous mutants had no detectable *Pitx2* expression in the IFT (*n*=26/27). In *Tbx6^−/−^;node-Tg* embryos, *Pitx2* expression was restored in the IFT in 8/22 (36%) embryos, all on the left side (Fig. 4).

The *node-Tg* transgene was also included in the previously described *Tbx6; Pkd2* cross (Fig. 2B; Table S1) where the proportion of *Tbx6* mutant embryos expressing left-sided *Pitx2* was 31% (8/26), which was further increased to 44% (17/39) in the presence of the transgene. Combining data from both series of *Gdf1* rescue experiments, 25/61 (41%) of *Tbx6* homozygous mutants with *Gdf1* restored in the perinodal region showed left-sided *Pitx2* expression (with a single embryo showing right-sided expression), compared with 8/53 (15%) showing left-sided expression without the transgene (Χ^2^=8, *P*<0.01). This indicates that perinodal Gdf1 can partially rescue asymmetric *Pitx2* expression in *Tbx6* mutant embryos and furthermore provides additional evidence for a functional leftward nodal flow in *Tbx6* mutant embryos as the rescued embryos showed predominantly left-sided *Pitx2* expression. As expected, the transgene had no apparent effect on the *Pkd2* phenotype.

## DISCUSSION

### Node formation and ciliogenesis

We investigated genes known to be involved in node and cilia development and found that expression of the homeobox gene *Noto*, a gene affecting node structure and cilia motility (Beckers et al., 2007), was delayed or irregular in half of the *Tbx6* mutants. However, the significance for this expression change in node and cilia formation is not clear as the expression of the downstream genes that mediate the effect on cilia, *Foxj1* and *Rfx3* (Alten et al., 2012; Bonnafe et al., 2004; Brody et al., 2000) is unaffected in mutants. Since *Noto* is a marker for the node, the irregular expression pattern observed may be simply a reflection of node morphological irregularities previously observed in *Tbx6* homozygous mutants (Hadjantonakis et al., 2008).

Previous work in Xenopus, zebrafish and mice implicated the non-canonical Wnt pathway in node and cilia development and left-right axis formation (Etheridge et al., 2008; Hamblet et al., 2002; Hashimoto et al., 2010; Kitajima et al., 2013; Minegishi et al., 2017; Oishi et al., 2006; Zhang and Levin, 2009). None of the non-canonical ligands or receptors we examined, however, differed in expression between mutants and controls.

### Asymmetric gene expression in the perinodal region and LPM

In our previous study, we postulated that the nodal flow was disrupted by the altered motility of the abnormal cilia in *Tbx6* mutants, accounting for a lack of Ca^+2^ signaling at the periphery of the node and subsequent disruption of left-right axis determination (Hadjantonakis et al., 2008). There was no expression of asymmetric genes in the LPM although we detected *Nodal* expression perinodally at a level too low to determine whether it was asymmetric. However, work by others has shown that a low level of perinodal *Nodal* expression is sufficient to initiate expression of left- (or right-) specific genes in the LPM (Brennan et al., 2002). Thus in the present study, we used an additional marker of asymmetry to determine whether the perinodal signal was asymmetric or random in *Tbx6* mutant embryos. We detected asymmetric right-sided expression of *Dand5*, a Nodal antagonist that is responsible for the robust asymmetric expression of *Nodal* and is the initiator of the asymmetric molecular cascade (Babu and Roy, 2013). Although the number of embryos examined was small, two additional lines of evidence support this conclusion. We found a proportion of *Tbx6* mutant embryos with sporadic *Pitx2* expression, which was on the left side in 8/8 embryos. Removal of *Pkd2*, a gene central to the detection of the nodal flow (Pennekamp et al., 2002; Yoshiba et al., 2012), from *Tbx6* mutants removed this left-bias, indicating that the nodal flow of *Tbx6* mutants is functional and is perceived by the perinodal crown cells. Finally, in the *Gdf1* rescue experiment, 25/26 of the rescued *Tbx6^−/−^;node-Tg* embryos showed left-sided *Pitx2* expression. Because *Tbx6* mutant embryos have randomized laterality, these results point to an additional role for *Tbx6* further downstream in the genetic cascade that prevents the asymmetric expression of *Nodal*, *Lefty2* and *Pitx2* in the left LPM of mutant embryos, a role separate from its effects on nodal cilia and perinodal expression of *Nodal*.

### Tbx6 is upstream of *Gdf1*

*Gdf1* expression is critical for the long-range action of *Nodal* in activating asymmetric expression of *Nodal* in LPM (Tanaka et al., 2007). We showed that *Tbx6* homozygous mutants do not express *Gdf1* in the perinodal region but that a *node-Tg* transgene that expresses *Gdf1* bilaterally in the perinodal region, could partially rescue *Pitx2* expression in the left LPM/IFT of *Tbx6* homozygous mutants. There are several possible reasons why rescue is not complete. First, in the transgene, *Gdf1* is driven by a node-specific *Nodal* enhancer, which was shown to partially rescue left-sided *Pitx2* expression in the LPM/IFT of *Gdf1* mutants where 2/6 embryos showed relatively normal *Nodal* expression and 4/6 showed a restricted pattern of expression (Tanaka et al., 2007) indicating that the transgene did not restore full function. Secondly, the low level of *Nodal* expression in the perinodal region of *Tbx6* mutants may compromise transduction of the signal, rendering rescue less likely.

The rescue experiment indicates that the absence of expression of left-specific genes in the LPM of *Tbx6* homozygous mutants is due to the lack of *Gdf1* perinodal expression. Although *Tbx6* regulates luciferase reporter activity from the *Gdf1* promoter, it does not do so through the putative *Tbx6* binding sites we identified within 2 kb of the *Gdf1* promoter region and thus *Tbx6* may regulate *Gdf1* expression through additional binding sites not detected in this analysis. The lack of a demonstrable genetic interaction in *Tbx6^+/−^;Gdf1^+/−^* and Tbx*6^rv/−^;Gdf1^+/−^* compound heterozygous embryos indicates that even a reduced amount of Tbx6 protein is sufficient to drive perinodal *Gdf1* expression.

In summary, the *Tbx6* mutation impinges on several different components of the left-right axis determination pathway by affecting development of the node and nodal cilia, decreasing the level of *Nodal* expression in the perinodal region, and eliminating asymmetric Ca^2+^ signaling at the node (Hadjantonakis et al., 2008). In this study, we have shown that in spite of these abnormalities, asymmetric gene expression indicative of a leftward nodal flow is still present and that *Tbx6* additionally regulates expression of perinodal *Gdf1*, resulting in failure of transduction of the asymmetric signal to the LPM in mutant embryos and ultimately resulting in the randomization of laterality phenotype. It is not clear whether disruptions of node and nodal cilia and lower perinodal *Nodal* expression contribute to the laterality phenotype of *Tbx6* mutants but it is interesting that Ca^2+^ signaling appears to be uncoupled from asymmetric gene expression at the node, indicating that disruptions of the nodal flow may differentially affect these two events.

## MATERIALS AND METHODS

### Mice, embryo collection and ISH

The null allele, *Tbx6^tm2Pa^* (referred to as *Tbx6^−^*) (Hadjantonakis et al., 2008) was maintained on a mixed 129 and ICR (Taconic) background. The null allele *Gdf1^tm1Sjl^*/J (B6;129- *Gdf1^tm1Sjl^*/J; The Jackson Laboratory, Bar Harbor, USA. Stock No. 004425; referred to as *Gdf1^−^*) is a targeted deletion of the protein coding region (Rankin et al., 2000). The *node-Tg* transgene contains *Gdf1* cDNA linked to *IRES-lacZ* under the control of the node-specific enhancer (NDE) of *Nodal*, which results in restricted expression of *Gdf1* bilaterally in the perinodal region (Tanaka et al., 2007). *Tbx6 rib vertebrae* (*Tbx6^rv^*/J) (B6.L-*Tbx6^rv^*/J; The Jackson Laboratory Stock No. 001052) is a hypomorphic allele (Watabe-Rudolph et al., 2002). Mice containing a null allele of *Pkd2* (referred to as *Pkd2^−^*) were obtained by crossing the *Pkd2^tm1.1Tjwt^*/J conditional allele [B6.129X1(Cg)- *Pkd2^tm1.1Tjwt^*/J; The Jackson Laboratory Stock No. 017292] with mice ubiquitously expressing *cre* recombinase (EIIaCre) and selecting mice with excision of exons 11-13 (*Pkd2^tm1.2Tjw^*) (Garcia-Gonzalez et al., 2010). All genotypes were maintained on mixed genetic backgrounds. To collect embryos for the epistasis experiment, a three-way cross was used to produce double-heterozygous *Tbx6; Pkd2* mice with or without the *Gdf1* transgene, which were mated together to produce embryos with all combinations of genotypes.

Embryos were recovered between E7.5 and E8.5 (E0.5 denotes noon of the day of a vaginal plug) in phosphate buffered saline (PBS) containing 0.2% albumin bovine serum (Sigma-Aldrich), and staged by morphology (Downs and Davies, 1993); some were scored for placement of the tail, placenta, vitelline vessels and the direction of heart looping. Yolk sacs were used for PCR genotyping with primers for *Tbx6*, *Tbx6^rv^*, *Gdf1*, *node-Tg* and *Pkd2* (Table S3). Alternatively, *Tbx6^−^* embryos were genotyped by fluorescence intensity which is proportional to the number of *Tbx6^−^* alleles (Hadjantonakis et al., 2008). Embryos for ISH were fixed overnight in 4% paraformaldehyde in PBS at 4°C, dehydrated in 100% methanol and stored at −20°C until being processed for ISH using antisense RNA probes (Wilkinson, 1998). All animal protocols were approved by the Columbia University Medical Center Institutional Animal Care and Use Committee.

### EMSA

A coupled *in vitro* transcription/translation system with rabbit reticulocyte lysate (TNT^®^ Promega, Madison, USA) was used to make full-length Tbx6 protein for DNA binding studies (White and Chapman, 2005). 1 μg of *Tbx6* cDNA cloned into a pET21a expression vector (Novagen, Sigma-Aldrich) was incubated in 50 μl of rabbit reticulocyte lysate for 1 h at 30°C. Fluorescent probes were generated by attaching a linker sequence 5′-GTAGAGACTCGT-3′ to the 3′ end of the reverse compliment of five putative *Tbx6* binding sites and mutant versions of each, created by mutating the core sequence of the T-box binding element. To create a double-stranded, fluorescently labeled DNA probe for EMSA, a fluorescently labeled oligonucleotide, Tbx6 linker: 5′-Cy5-GTAGAGACTCGT-3′, was annealed to the linker sequence on each of the reverse compliment oligonucleotides using KLENOW polymerase to fill in the remaining sequence. Three microliters of lysate containing either the Tbx6 expression construct or empty vector was incubated with 0.3 ng of each fluorescently labeled probe in binding buffer (20 mM HEPES, pH 7.5, 50 mM KCl, 5 mM MgCl_2_, 10 µM ZnCl_2_, 6% glycerol, 200 µg of bovine albumin/ml, and 50 µg of poly(dI-dC)·poly(dI-dC)/ml) (Gebelein and Urrutia, 2001) for 20 min at room temperature. Each binding reaction was then loaded onto a 4% polyacrylamide gel and run at 120 V at 4°C. Fluorescent probe alone was loaded as a negative control. The gel was then vacuum dried and imaged using a Typhoon TRIO variable mode imager (Amersham Biosciences, Little Chalfont, UK).

### Cell culture and luciferase assays

NIH-3T3 cells (ATCC) were cultured in DMEM (Gibco) with 10% FBS (HyClone, Thermo Fisher Scientific), 1% Penicillin/Streptomycin (Gibco), 1% non-essential amino acids (Gibco), 1% sodium pyruvate (Gibco). Cells were split at 80% confluency and plated at a concentration of 200,000 cells per ml for transfection with a luciferase reporter containing the promoter region of *Gdf1*, a luciferase reporter containing mutated putative *Tbx6* binding sites, and *Tbx6* cDNA cloned in-frame into the expression vector pCMV-Tag2a that inserts a FLAG tag at the 5′ end of Tbx6 protein. Transfection was achieved by PEI-based transfection (Ehrhardt et al., 2006). Cells were collected after 48 h and lysed in cold NP40 Vanadate lysis buffer containing protease inhibitor cocktail (Sigma-Aldrich) and 1 mM phenylmethylsulfonyl fluoride for 20 min. Cells were centrifuged and protein in the supernatant was quantified by Bradford reagent (Bio-Rad) and used for western blotting. Then, 10 μg of protein/sample/lane was run on a 4% polyacrylamide gel and transferred onto polyvinylidene difluoride membrane. Western Blots were blocked in 4% milk for 30 min at room temperature and incubated with mouse anti-FLAG antibody at 1:200 (Sigma-Aldrich) overnight at 4°C. Blots were washed with PBS containing 0.1% Tween-20 and anti-mouse horseradish peroxidase (The Jackson Laboratory) was added at a concentration of 1:10,000 for 1 h. Blots were visualized with ECL Substrate (Thermo Fisher Scientific).

## Supplementary Material

Supplementary information
