# Supplementary material for: Tbx6 controls left-right asymmetry through regulation of Gdf1
Source: Biol Open. 2018 Apr 12;7(5):bio032565. doi: 10.1242/bio.032565 (PMC5992533; doi:10.1242/bio.032565)
Supplement: Supplementary information [file biolopen-7-032565-s1.pdf]

Supplementary Table 1. *Pitx2* expression in the lateral plate mesoderm or inflow tract of the heart (LPM/IFT) in embryos at E8.5 from crosses of mice heterozygous for *Tbx6* and *Pkd2* and hemizygous for the *node-Tg* transgene (Tg). Wild type includes embryos homozygous wild type or heterozygous for one or both genes. Frequencies are not mendelian as not all wild type embryos were processed. Data is presented in graphic form in Figure 2.

Genotype				n	Left	Right	Bilateral	None
Wild type	*Tbx6*	*Pkd2*	*Gdf1* Tg					
	+/+	+/+	-	5	4			1
	+/+	+/-	-	7	7			
	+/-	+/+	-	2	2			
	+/-	+/-	-	12	12			
Wild type with *Gdf1* transgene	+/+	+/+	+	5	5			
	+/+	+/-	+	7	7			
	+/-	+/+	+	4	4			
	+/-	+/-	+	17	16			1
Tbx6 null	-/-	+/+	-	10	4			6
	-/-	+/-	-	16	4			12
Tbx6 null with *Gdf1* transgene	-/-	+/+	+	10	5			5
	-/-	+/-	+	29	12	1		16
Pkd2 null	+/+	-/-	-	5	1		1	3
	+/-	-/-	-	23	1	1	6	15
Pkd2 null with *Gdf1* transgene	+/+	-/-	+	1				1
	+/-	-/-	+	20	2	4	5	9
Double null	-/-	-/-	-	10	1		1	8
Double null with *Gdf1* transgene	-/-	-/-	+	9		1		8
TOTAL				192				

Biology Open • Supplementary information

Biology Open (2018): doi:10.1242/bio.032565: Supplementary information

Supplementary Table 2. Sequences of *Tbx6* putative bindings sites and wild type and mutated EMSA DNA probes.

**Tbx6 binding site #**	**Location relative to *Gdf1 t*ranscriptional start site**	***Tbx6* DNA binding site sequence**	**Wild type EMSA DNA probe**	**Mutant EMSA DNA probe**
1	-1522	AGGTGTGACAGC	GGAACTGCGAGCTGTCACACCTCTCCAGACCCACGAGTCTCTAC	GGAACTGCGAGCTGTC*TTTTAG*CTCCAGACCCACGAGTCTCTAC
2	-1356	AGGTGAGAGGGC	TAGCTCTCTAGCCCTCTCACCTACACCATTTAACGAGTCTCTAC	TAGCTCTCTAGCCCTCT*TTTAG*ACACCATTTAACGAGTCTCTAC
3	-836	AGGTTTTAAATA	TCCTCCATCAGTATTTAAAACCTTGGATGGCTCAACGAGTCTCTAC	TCCTCCATCAGTATTTA*TTTTAG*TGGATGGCTCAACGAGTCTCTAC
4	-544	AGGAGTTAGTAA	TAGCCTGATCCTTACTAACTCCTCCAGCCATATAACGAGTCTCTAC	TAGCCTGATCCTTACTATTTTAGCCAGCCATATAACGAGTCTCTAC
5	989	AGGTGCTGACTG	CGCATCTGACCAGTCAGCACCTTGGCTGCGAAACGAGTCTCTAC	CGCATCTGACCAGTCATTTTAGTGGCTGCGAAACGAGTCTCTAC

Biology Open • Supplementary information

Supplementary Table 3. Primer sequences used for genotyping mice and embryos (Hadjantonakis et al., 2008; Rankin et al., 2000; Tanaka et al., 2007; Watabe-Rudolph et al., 2002).

Genotype	Primer Name	Primer Sequence
Tbx6	T6koGS1	ATTGCACGCAGGTTCTCCGG
	T6koGS2	GTCACGACGAGATCCTCGCC
	T6met	GGGAGAATGGGATCCAGG
	T6ln	TACCATCCACGAGAGTTGTAC
Tbx6rv	Rv1b	GTGTCTGGCGTATCAGCTCA
	Rv2a	CTCGCAGCTTCACTAGTCC-
Gdf1	oIMR183	GTTGCGGCTGGAGGCTGAGAG
	oIMR184	CCCACTGGACCAACTTCTACC
	oIMR185	CCACTGCAGCCTGTGGGCGC
	oIMR186	GGAAGACAATAGCAGGCATGCTGG
Node-Tg	ZS-6	GCACGGTTACGATGCGCCCATCTACACCAACGT
	ZA-6	ACGGCAAACGACTGTCCTGGCCGTAACCGACC
Pkd2	Pkd2a	CCTTTCCTCTGTGTTCTGGGGAG
	Pkd2c	CTGACAGGCACCTACAGAACAGTG

Biology Open • Supplementary information

Supplemental Fig. 1

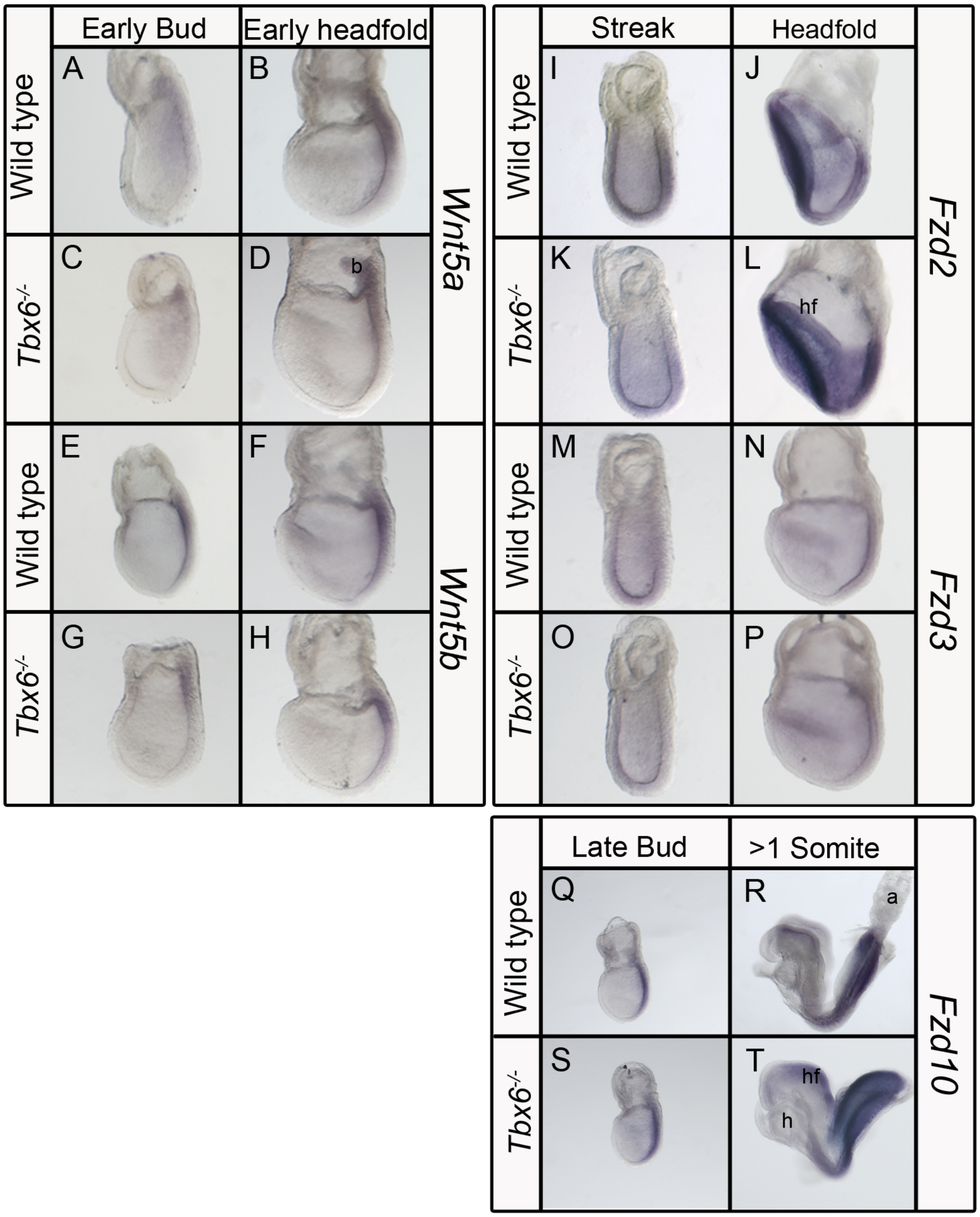

Supplemental Figure 1. Expression of components of the non-canonical Wnt signaling pathway in wild type and *Tbx6* homozygous mutants is similar during early development. A-H. Expression of non-canonical Wnt signaling ligands *Wnt5a* and *Wnt5b* in the posterior side of the embryo between the early allantoic bud and early headfold stages (Gavin et al., 1990. *Genes Dev.* **4**, 2319-2332). **I-T.** Expression of the receptors *Fzd2*, *Fzd3* and *Fzd10* at the stages indicated. *Fzd2* and *Fzd3* are expressed in the epiblast (Wang et al.,1996. *J. Biol. Chem.* **271**, 4468-4476). *Fzd10* expression is in the posterior of the embryo extending to the distal tip at the late allantoic bud stage and later in the headfolds, somites and presomitic mesoderm (Kim et al., 2001. *Mech. Dev.* **103**, 167-172). All panels are left lateral views. a, allantois; b, allantoic bud; h, heart; hf, headfolds.

Biology Open • Supplementary information

Supplemental Fig 2

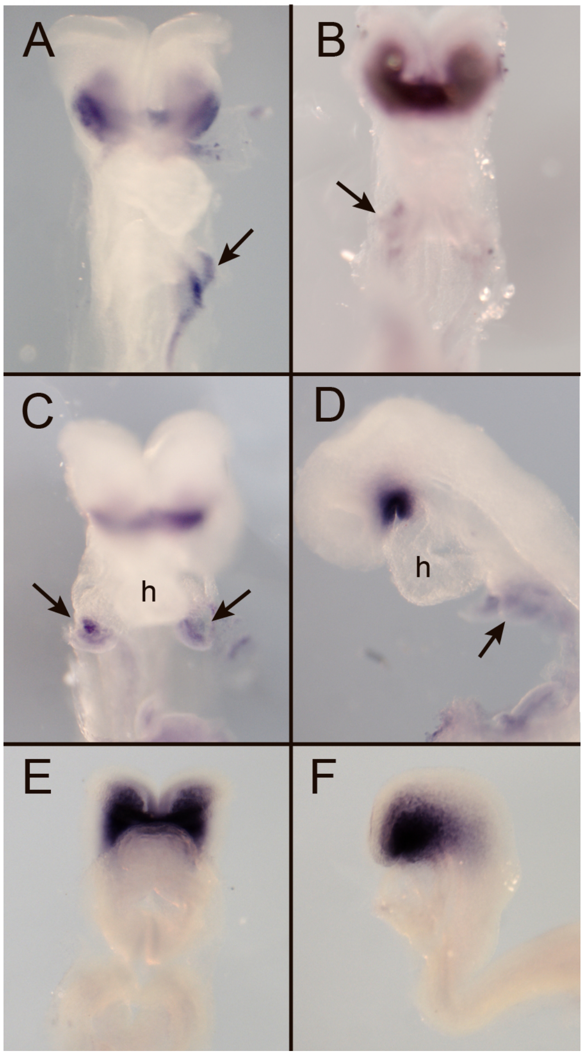

Supplemental Figure 2. *Pitx2* expression in representative E8.5 embryos from the three-way *Tbx6*, *Pkd2*, node *Gdf1* transgene cross. *Pitx2* is expressed in the head region of all embryos. Arrows indicate LPM expression. **A.** A *Tbx6* mutant with the transgene showing left-sided expression. **B.** A double null mutant with the transgene showing-right sided expression. **C, D.** A double null embryo without the transgene showing bilateral expression. Note the ventrally-looped heart (h) in D. **E, F.** A double null embryo without the transgene showing no expression in the LPM. D and F are left side views; other panels are ventral views.

Biology Open • Supplementary information
